# Supplementary material for: Genome Comparisons between Botrytis fabae and the Closely Related Gray Mold Fungus Botrytis cinerea Reveal Possible Explanations for Their Contrasting Host Ranges
Source: J Fungi (Basel). 2024 Mar 14;10(3):216. doi: 10.3390/jof10030216 (PMC10971195; doi:10.3390/jof10030216)
Supplement: Supplementary file 1 [file jof-10-00216-s001.zip › Table S9.pdf]

Table S9. Expression levels of strain-specific genes in BfabG12 and BcB0510.

	Strain	All genes	Specific genes	Truncated in BfabG12	Truncated in BcB0510
Median RPKM values	BfabG12	20.00	1.15	1.00	2.09
	BcB0510	16.83	1.68	1.85	0.91
Expressed genes[a]	BfabG12	8962 (78.7%)	52 (35.9%)	125 (31.2%)	35 (48.6%)
	BcB0510	9032 (77.2%)	226 (48,0%)	192 (47.9%)	23 (31.9%)

[a] RPKM values ≥ 2
