# Supplementary material for: Genome Comparisons between Botrytis fabae and the Closely Related Gray Mold Fungus Botrytis cinerea Reveal Possible Explanations for Their Contrasting Host Ranges
Source: J Fungi (Basel). 2024 Mar 14;10(3):216. doi: 10.3390/jof10030216 (PMC10971195; doi:10.3390/jof10030216)
Supplement: Supplementary file 1 [file jof-10-00216-s001.zip › Table S10.pdf]

Table S10. Non-canonical introns in *B. fabae* G12 genes.

Gene	Predicted function	Position	5´ border	3´ border	Same in BcB0510?
Bfab01g03590.2	Unknown	1:1217963	Canonical	AAT	Yes
Bfab01g05920.1	Synaptojanin 2 (endocytosis)	1:2081354	GAAAGT	Canonical	Yes
Bfab01g10140.1	Flavin nucleotide binding protein	1:3568200	Canonical	CGG	No
Bfab02g03620.1	GAL4-like transcription factor	2:1334384-436	ATAAGT	TAC	Yes
Bfab02g04040.1	Unknown	2:1465304-54	GAGTGA or TGAGTG [a]	TAT or TTA [a]	Yes
Bfab02g06250.2	Tubulin binding cofactor C	2:2370651-703	ACAGGG (or CAGGGG) [a]	TCG (or CGA) [a]	Yes
Bfab03g01840.1	Unknown	3:687271	Canonical	TCG	No
Bfab03g06466.1	MFS multidrug transporter	3:2326885	Canonical	TGG	---
Bfab04g00640.1	Polyketide synthase	4:309099	GGTAAG	Canonical	No
Bfab04g04620.1	WD40 domain protein	4:1652470-517	ATAAGT (or TAAGTT) [a]	CAC (or ACA) [a]	Yes
Bfab04g05830.1	Unknown	4:2076054-105	ATATGT	TAC	Yes
Bfab04g05860.1	Acid phosphatase	4:2082338	Canonical	CCC	No
Bfab07g05890.1	CTP synthase	7:2264270-326	ATATGT	AAC	Yes
Bfab09g06160.1	Vacuolar ATP synthase subunit E	9:2488010-65	ATACGT (or TACGTC) [a]	AAC (or ACA) [a]	Yes
Bfab15g03830.1	Unknown	15:1371490	Canonical	TAT	Yes
Bfab16g05090.1	Signal transduction histidine kinase	16:2108435	Canonical	CGG	Yes

[a] Cannot be distinguished by comparison of RNAseq reads and BfabG12 or BcB0510 genomic DNA
