# Supplementary material for: Genome Comparisons between Botrytis fabae and the Closely Related Gray Mold Fungus Botrytis cinerea Reveal Possible Explanations for Their Contrasting Host Ranges
Source: J Fungi (Basel). 2024 Mar 14;10(3):216. doi: 10.3390/jof10030216 (PMC10971195; doi:10.3390/jof10030216)
Supplement: Supplementary file 1 [file jof-10-00216-s001.zip › Table S13.pdf]

Table S13. Primers used in this study.

Name	Sequence (5' – 3')	Gene, purpose
pNAH_fw	GATGGCCCTTCCCATTTAC	GFP-hyg-expressing construct, amplified from pNAH-OGG
pNAH_rev	AGATGCTGCTGGCAAGGTTAC	
PZChr7Gap-F	GTATCTGAAAGGGAGGAGGATGTC	Gap closure in Bfab7
PZChr7Gap-R	CAAACTTGGCCACATTCAAACAAG	
PZChr7Gap1-F	TGAAGTCTGTTACGAGAGTG	Gap closure in Bfab7
PZChr7Gap1-R	GCTTTTCTCTCATCCAATCA	
PZ040	AGAGCTGATTTGAAGACATGC	Gap closure in Bfab7
PZChr12Gap1F	GAAACGAAGCTCTAGCCATCAATC	Gap closure in Bfab12
PZChr12Gap1R	AGGGTTATGAAGACTCACTCCAAC	
PZChr12Gap2F	TTATCTTCCTCGAGCTCGTTACTG	Gap closure in Bfab12
PZChr12Gap2R	GGTACACTCAAGTCAAGTTGTTCG	
PZChr12Gap3F	CTCGAAGCTTTCAATCCTCGAATC	Gap closure in Bfab12
PZChr12Gap3R	GTCGTCTAGTCGTTTGTTCCAATC	
PZChr12Gap4F	CAGTACGAAGCGAAGTGTGTGG	Gap closure in Bfab12
PZChr12Gap4R	GATACCATGAATGATGTACACG	
PZChr12Gap5F	GGCATTCGTGCTCAACGCGAG	Gap closure in Bfab12
PZChr12Gap5R	TTGAAACTTATATTTCTAATC	
PZChr12Gap6F	ACAGCGGTAAGTTTGCCGCAC	Gap closure in Bfab12
PZChr12Gap6R	CACCCTCAAGCGGGCTCGGAC	
PZChr12Gap7F	ATACGTGGGGGATGGATGTGG	Gap closure in Bfab12
PZChr12Gap7R	GATGGATGACGCATTGATTGA	
PZChr12Gap8F	TCAAGTTCATTGTCAGTGTC	Gap closure in Bfab12
PZChr12Gap8R	GTATACGACTCATCGCTTTTC	
PZChr12Gap9F	TGACTGTGCCACAACGGCAT	Gap closure in Bfab12
PZChr12Gap9R	CTTGATCTTCTCCTACGTTC	
PZChr12Gap10F	TTCTGGACCCAGTTCGTTCC	Gap closure in Bfab12
PZChr12Gap10R	TGCGCGATGGGGAGTGCAGC	
