# Supplementary material for: Genome Comparisons between Botrytis fabae and the Closely Related Gray Mold Fungus Botrytis cinerea Reveal Possible Explanations for Their Contrasting Host Ranges
Source: J Fungi (Basel). 2024 Mar 14;10(3):216. doi: 10.3390/jof10030216 (PMC10971195; doi:10.3390/jof10030216)
Supplement: Supplementary file 1 [file jof-10-00216-s001.zip › Table S1.pdf]

Table S1. Inversions in *B. fabae* G12 relative to *B. cinerea* B05.10.

No	Position in BfabG12	Size [bp]	% GC	Next to BfabG12 gene deletion
1	1:31126-501811	470685	42,3	Bcin01g01340-01420
2	1:674783-679128	4345	19,9[c]	no
3	2:1060476-1062046	1570	41,8	no
4	2:1602844-1609648	6804[b]	42,9	no
5	3:2060364-2087964	27600	43,5	no
6	3:2088517-2105387	16870	42,3	no
7	3:2105686-2141552	35866	44,6	no
8	3:3422717-3430397	7680	33,3	Bcin03g09310
9	4:2482932-2603674	120742	20,6	no
10	6:21432-144240	122808	28,3	Bcin06g00010-00040; 00210
11	6:589577-610883	21306	42,8	Bcin06g01480; 01540-01560
12	6:1030079-1151491	121412	42,0	no
13	7:379052-380108	1056	40,1	Bfab07g01020 (trunc.)
14	7:2453601-2454672	1071	42,3	no
15	8:268085-268377	292	42,7	no
16	8:958906-1077804	118898	44,2	Bfab08g02180, 02200
17	8:1936595-1938531	1936	42,5	no
18	9:2232229-2275285[a]	43056	31,3	Bfab09g05720, 05730
19	10:90080-90290	210	42,6	no
20	10:735145-741787	6642	40,7	no
21	11:229923-231363	1440	21,4	no
22	11:760537-761268	731	42,2	no
23	11:2115491-2115713	222	43,6	no
24	13:89320-106994	17674	23,1	no
25	14:332075-333371	1296	42,6	Bfab14g00870, 00880 (trunc.)
26	14:366707-384935	18228	41,8	no
1	14:722086-728926[b]	6840	42,6	no
28	15:1867072-1875953	8881	38,1	no
29	16:5278-8957	3679	34,3	no
30	16:455713-457407	1694	43,5	no

[a] Many smaller inversions

[b] Contains two retrotransposon-derived genes

[c] Numbers in red indicate the presence of AT-rich regions
