# Supplementary material for: Genome Comparisons between Botrytis fabae and the Closely Related Gray Mold Fungus Botrytis cinerea Reveal Possible Explanations for Their Contrasting Host Ranges
Source: J Fungi (Basel). 2024 Mar 14;10(3):216. doi: 10.3390/jof10030216 (PMC10971195; doi:10.3390/jof10030216)
Supplement: Supplementary file 1 [file jof-10-00216-s001.zip › Table S2.pdf]

Table S2. Occurrence of TE remnants in AT-rich regions.

	BfabG12	BcB0510
Total no of AT regions	483	281
AT regions with TE remnants	358	152
AT regions with Ty1-RE	167	36
AT regions with Ty3-RE	136	64
AT regions with LINE-RE	0	5
AT regions with TIR-Flipper	178	79
AT regions with TIR-Mariner	4	6
