# Supplementary material for: Genome Comparisons between Botrytis fabae and the Closely Related Gray Mold Fungus Botrytis cinerea Reveal Possible Explanations for Their Contrasting Host Ranges
Source: J Fungi (Basel). 2024 Mar 14;10(3):216. doi: 10.3390/jof10030216 (PMC10971195; doi:10.3390/jof10030216)
Supplement: Supplementary file 1 [file jof-10-00216-s001.zip › Table S3.pdf]

Table S3. Location of intact TE/RE-derived genes in BfabG12.

Type of TE	Coordinates	Gene ID	Size (aa)	Terminal repeats	close to AT-region?
Boty	2:1603508-1608944	Bfab02g04286	1622	584 bp LTR	within AT-region
Boty	6:1361857-1368515	Bfab06g03515	1622	584 bp LTR	next to AT-region
Boty	8:46656-53314	Bfab08g00115	1622	584 bp LTR	next to AT-region
Boty	9:301433-306869	Bfab09g00886	1622	584 bp LTR	no
Boty	14:722822-728258	Bfab14g01776	1622	584 bp LTR	within AT-region
Ty1-Copia	8:786449-779660	Bfab08g01765	1672	184 bp LTR	within AT-region
TIR-Flipper-1	5:1777875-1776014	Bfab05g04885	572	44 bp TIR	no
TIR-Flipper-1	7:1080292-1078431	Bfab07g02925	572	44 bp TIR	within AT-region
TIR-Flipper-1	8:1725030-1723153	Bfab08g03825	572	44 bp TIR	within AT-region
TIR-Flipper-1	14:2071197-2073054	Bfab14g05225	597	44 bp TIR	within AT-region
TIR-Flipper-2	15:1769760-1768120	Bfab15g1602550	547	44 bp TIR	next to AT-region
