# Supplementary material for: Genome Comparisons between Botrytis fabae and the Closely Related Gray Mold Fungus Botrytis cinerea Reveal Possible Explanations for Their Contrasting Host Ranges
Source: J Fungi (Basel). 2024 Mar 14;10(3):216. doi: 10.3390/jof10030216 (PMC10971195; doi:10.3390/jof10030216)
Supplement: Supplementary file 1 [file jof-10-00216-s001.zip › Table S6.pdf]

Table S6. Results of low-resolution genome sequencing of *Botrytis* spp.

Botrytis species	*B. fabae*			*B. pseudo-cinerea*	*B. calthae*
Strain	Bf611	Bf612	11002	VD110	MUCL1089
Coverage (assemblies)	27x	45x	29.3x	29.0x	27.6x
N50 scaffolds (kb)	24.0	24.1	98.0	171.5	150.2
Assembly size (Mb)	41.0	40.6	40.79	40.61	44.03
No of predicted genes	11,258	11,433	11,743	11,703	11,791
GC content (total) CL	41.0%	40.6%	42.8%	42.7%	41.3%
Median intron length	n.d.	n.d.	59 nt	59 nt	59 nt
Reference	Lee et al. (2020)		This work		
