# Supplementary figures and images for: Genome Comparisons between Botrytis fabae and the Closely Related Gray Mold Fungus Botrytis cinerea Reveal Possible Explanations for Their Contrasting Host Ranges
Source: J Fungi (Basel). 2024 Mar 14;10(3):216. doi: 10.3390/jof10030216 (PMC10971195; doi:10.3390/jof10030216)

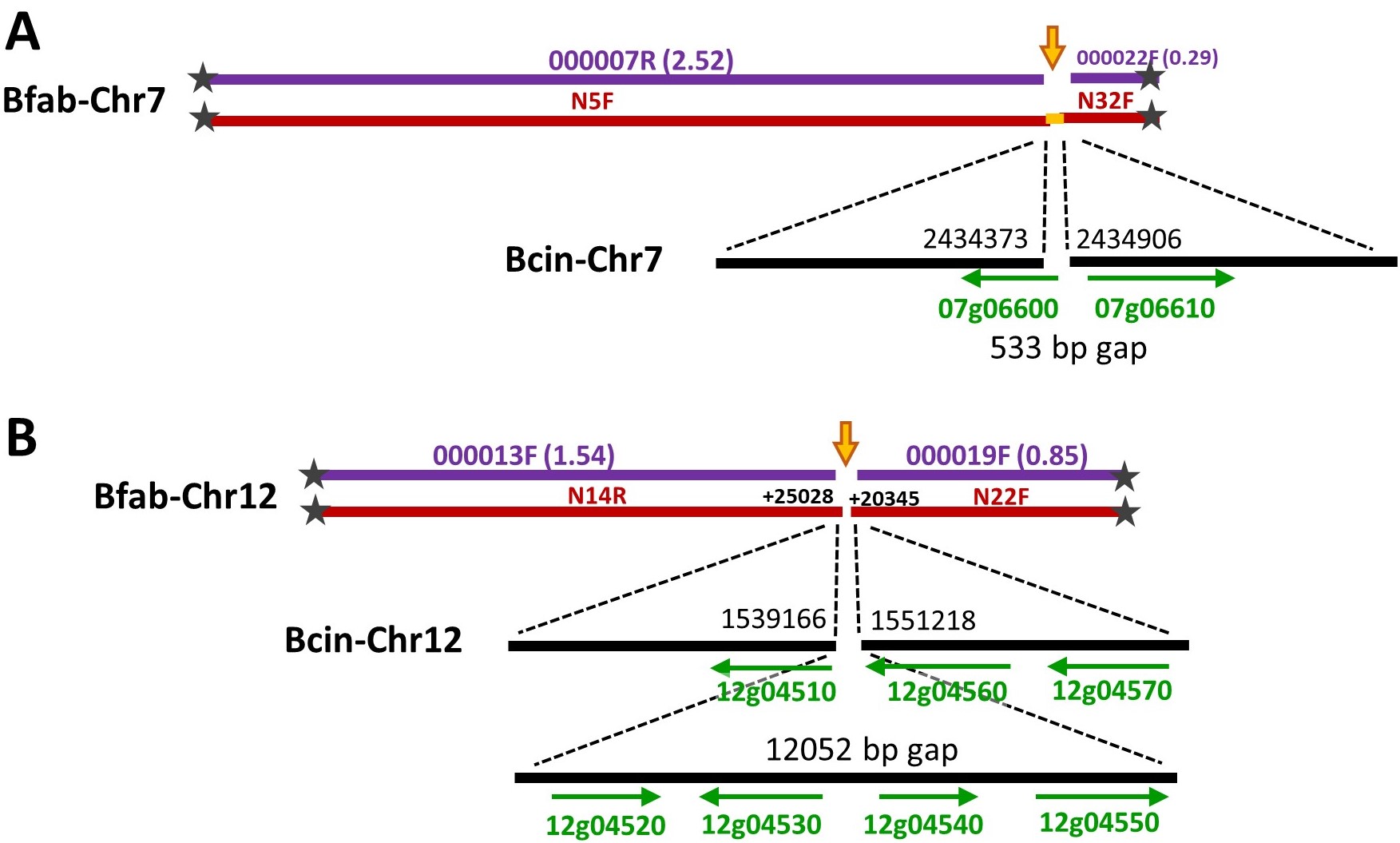

Supplement: Supplementary file 1 [file jof-10-00216-s001.zip › Figure S1.jpg]

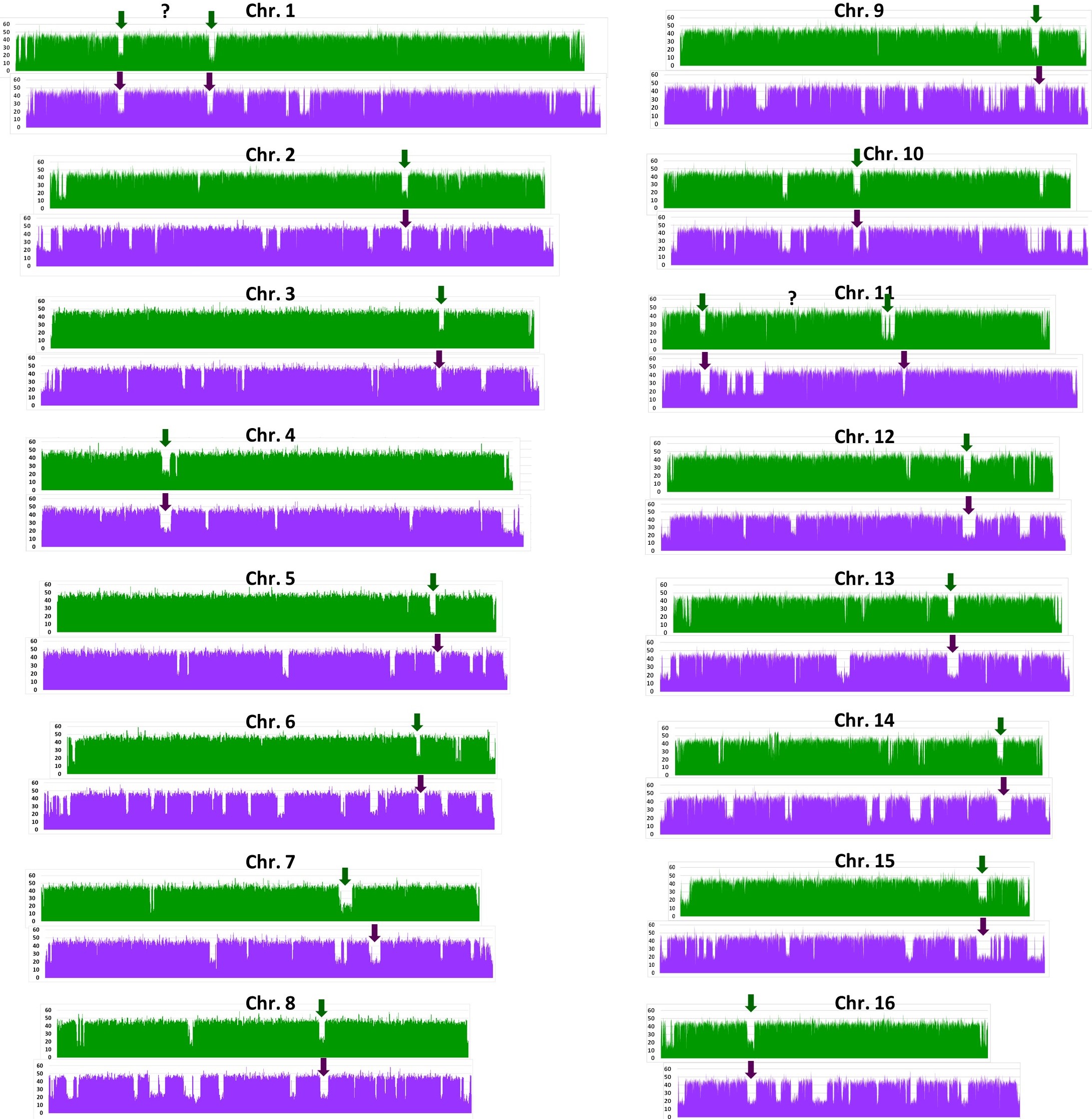

Supplement: Supplementary file 1 [file jof-10-00216-s001.zip › Figure S2.jpg]

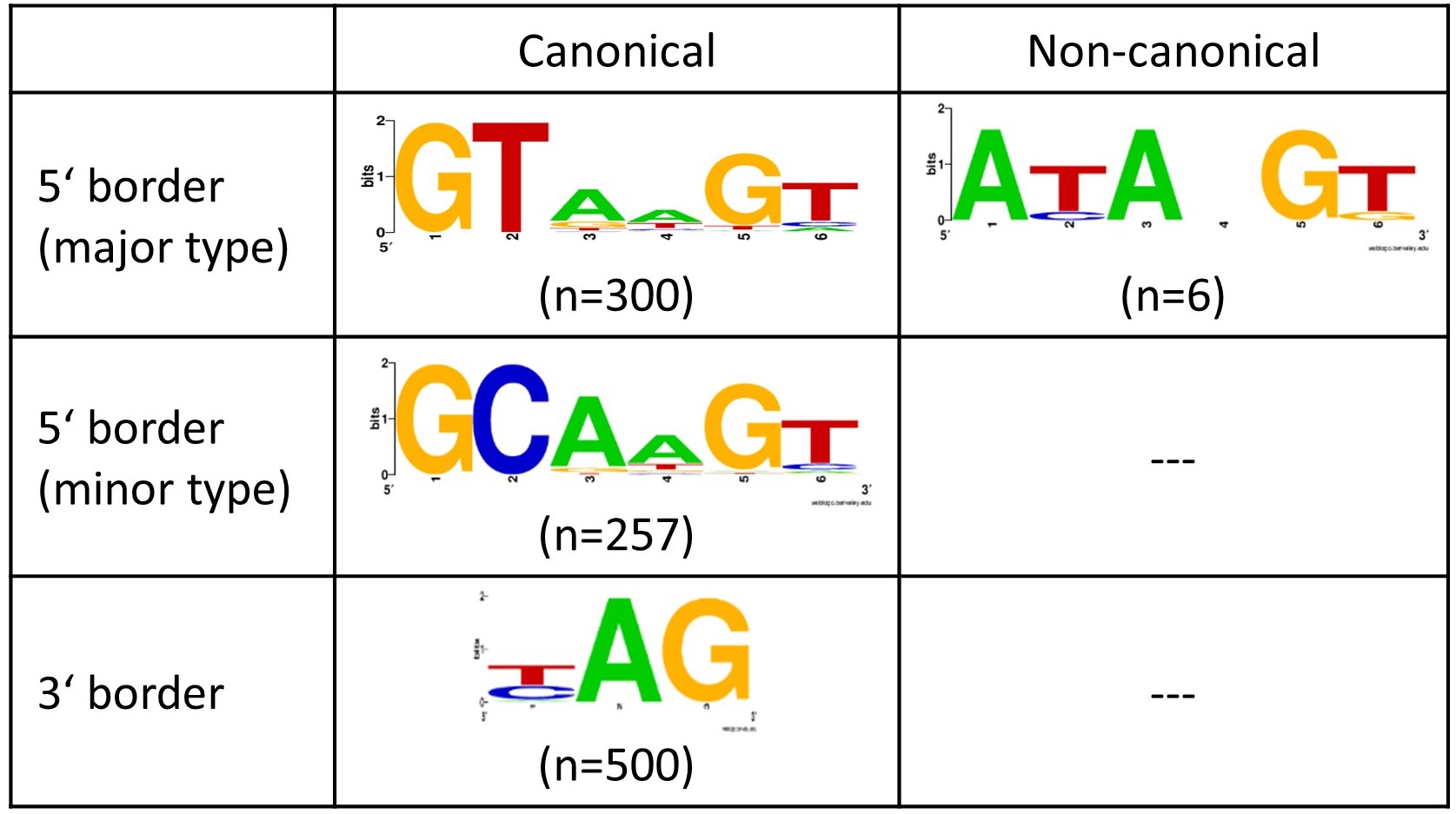

Supplement: Supplementary file 1 [file jof-10-00216-s001.zip › Figure S3.jpg]

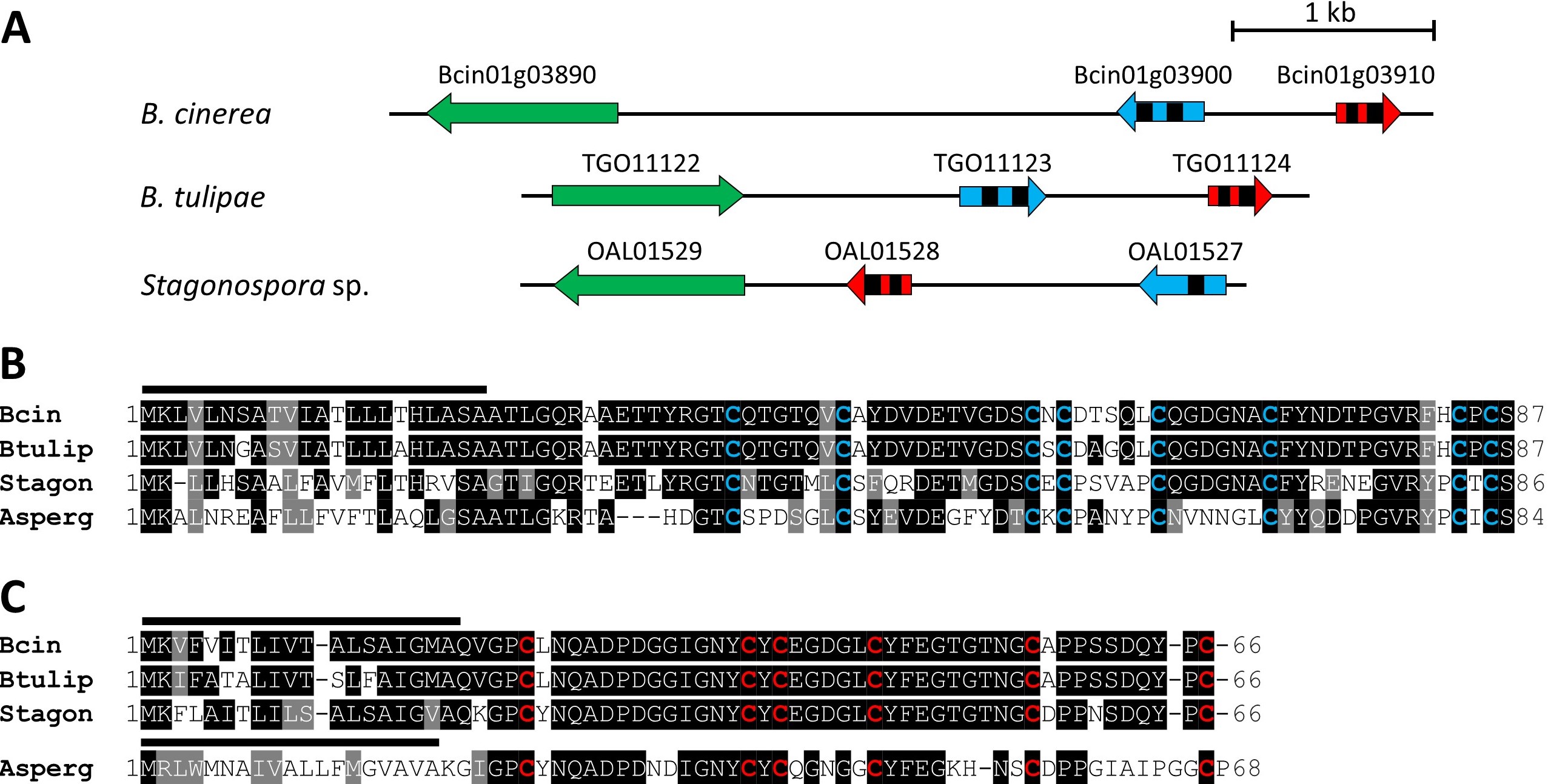

Supplement: Supplementary file 1 [file jof-10-00216-s001.zip › Figure S4.jpg]
